# Polymeric Microspheres/Cells/Extracellular Matrix Constructs Produced by Auto-Assembly for Bone Modular Tissue Engineering

**DOI:** 10.3390/ijms22157897

**Published:** 2021-07-23

**Authors:** Bartosz Mielan, Daniela M. Sousa, Małgorzata Krok-Borkowicz, Pierre Eloy, Christine Dupont, Meriem Lamghari, Elżbieta Pamuła

**Affiliations:** 1 Department of Biomaterials and Composites, Faculty of Science and Ceramics, AGH University of Science and Technology, Al. Mickiewicza 30, 30-059 Kraków, Poland; barmie@agh.edu.pl; 2 Instituto de Investigação e Inovação em Saúde (i3S), Rua Alfredo Allen 208, 4200-135 Porto, Portugal; danisous@ineb.up.pt (D.M.S.); lamghari@ineb.up.pt (M.L.); 3 Instituto de Engenharia Biomédica (INEB), Universidade do Porto, 4200-135 Porto, Portugal; 4 Faculté des Bioingénieurs Institut de la Matière Condensée et des Nanosciences, Université Catholique de Louvain, Place L. Pasteur 1, 1348 Louvain-la-Neuve, Belgium; pierre.eloy@uclouvain.be (P.E.); christine.dupont@uclouvain.be (C.D.); 5 Instituto de Ciências Biomédicas Abel Salazar (ICBAS), Universidade do Porto, 4050-313 Porto, Portugal

**Keywords:** microspheres, cell carriers, modular tissue engineering, cell adhesion, cell differentiation

## Abstract

Modular tissue engineering (MTE) is a novel “bottom-up” approach to create engineered biological tissues from microscale repeating units. Our aim was to obtain microtissue constructs, based on polymer microspheres (MSs) populated with cells, which can be further assembled into larger tissue blocks and used in bone MTE. Poly(L-lactide-*co*-glycolide) MS of 165 ± 47 µm in diameter were produced by oil-in-water emulsification and treated with 0.1 M NaOH. To improve cell adhesion, MSs were coated with poly-*L*-lysine (PLL) or human recombinant collagen type I (COL). The presence of oxygenated functionalities and PLL/COL coating on MS was confirmed by X-ray photoelectron spectroscopy (XPS). To assess the influence of medium composition on adhesion, proliferation, and osteogenic differentiation, preosteoblast MC3T3-E1 cells were cultured on MS in minimal essential medium (MEM) and osteogenic differentiation medium (OSG). Moreover, to assess the potential osteoblast–osteoclast cross-talk phenomenon and the influence of signaling molecules released by osteoclasts on osteoblast cell culture, a medium obtained from osteoclast culture (OSC) was also used. To impel the cells to adhere and grow on the MS, anti-adhesive cell culture plates were utilized. The results show that MS coated with PLL and COL significantly favor the adhesion and growth of MC3T3-E1 cells on days 1 and 7, respectively, in all experimental conditions tested. On day 7, three-dimensional MS/cell/extracellular matrix constructs were created owing to auto-assembly. The cells grown in such constructs exhibited high activity of early osteogenic differentiation marker, namely, alkaline phosphatase. Superior cell growth on PLL- and COL-coated MS on day 14 was observed in the OSG medium. Interestingly, deposition of extracellular matrix and its mineralization was particularly enhanced on COL-coated MS in OSG medium on day 14. In our study, we developed a method of spontaneous formation of organoid-like MS-based cell/ECM constructs with a few millimeters in size. Such constructs may be regarded as building blocks in bone MTE.

## 1. Introduction

One of the main issues in traditional tissue engineering (TTE) is limited diffusion of nutrients and waste removal, which are impeded in the center of the scaffolds [1]. Modular tissue engineering (MTE) is a novel concept that differs from the TTE approach based on centimeter size scaffolds, cells, and signaling molecules. MTE uses small building blocks, which can be organized into more complex and functional tissue-like constructs, sometimes made of different types of cells, e.g., those involved in osteogenesis and neovascularization [2,3]. The MTE approach may provide conditions allowing the cells to build and remodel all components, i.e., microscaffolds, extracellular matrix (ECM), and other cells, because cells may arbitrarily organize their surroundings, as happens in vivo. In TTE, it is rather impossible because macroscopic scaffolds have strictly defined shape and porosity. In MTE, it is not a porosity of the material that determines where cells grow but the cells themselves [4]. The fact that natural organs are made of small, three-dimensional (3D), well-organized, repeating units makes the MTE approach more biomimetic than TTE [3,4,5] and allows very complex tissues such as in the liver or trachea to be obtained [2]. Additionally, MTE approaches make it possible to obtain complex cell–material constructs by defining the organization of such modules with numerous additive manufacturing methods such as bioprinting [2].

Microspheres (MSs) may be regarded as a kind of microscaffold that can be used in MTE. MSs provide high relative surface area, and thus, access to nutrients from the medium and removal of the waste products are facilitated [6]. Cells may grow on MSs in three dimensions, which creates conditions that are more similar to natural ones [7]. Thus, for this reason, cells on MSs are more resistant to losing their phenotype, which may occur in 2D cell cultures [8,9]. Scaffolds in a form of MS can play the role of injectable cell carriers [10] to fill in defects with low invasiveness techniques, which is less harmful to the patient [11,12]. MSs intended for cell culture reported in the literature had different diameters: 80–90 µm [13], 74–150 µm [14], 100–250 µm [15], and 125–250 µm [16], but in a majority of studies, their size was higher than 100 µm [15,16,17,18]. MSs can be also used as cell carriers and form tissue in situ, e.g., for cartilage repair [2].

MSs can be made of polymeric biomaterials, including bioresorbable poly(L-lactide-*co*-glycolide) (PLGA) [11]. PLGA is an aliphatic polyester used for many years in a number of medical applications owing to its well-established biocompatibility. It is usually synthesized by ring-opening polymerization of L-lactide and glycolide. This makes it possible to control the degradation time of PLGA by LA:GA ratio in the polymer composition. PLGA has been used to produce scaffolds for tissues of every type [19] due to its mechanical properties and ease of processing [14]. It is also a good candidate for MS manufacturing.

Despite numerous PLGA advantages due to its hydrophobic character and chemical inertness, there is a need to improve cell attachment by modification with substances that support cell adhesion [14]. Gelatin [20], poly-*L*-lysine (PLL) [21], chitosan, or proteins with cell-adhesive arginine–glycine–aspartic acid (RGD) domains [14,22] such as collagen [23] have been used to modify different polymeric substrates. Collagens are a wide group of proteins made of three α-chains, which form triple-helical and non-triple-helical domains. Integrins α1β1 can recognize specific domains in collagen, resulting in the creation of focal adhesions plaques, which are responsible for the cell adhesion process [24,25]. PLL is a polypeptide made of L-lysine—a positively charged amino acid due to the presence of amino groups in this molecule. Due to the negative charge of the cell membrane, cells interact with positively charged PLL molecules, which results in improved cell adhesion to surfaces modified with PLL. Additionally, the high biocompatibility and antibacterial properties of PLL made it a commonly applied modifier of the surfaces used for cell cultures [11,26,27].

The aim of this study was to manufacture PLGA MS, modify their surface with adhesion-supportive molecules such as PLL and collagen (COL), and compare their efficiency in stimulating preosteoblast MC3T3-E1 cells differentiation and matrix production. As shown in the literature, cells cultured on MSs in 3D conditions have a lower potential to dedifferentiate [8,9]. In this experiment, we hypothesize that it may work in an opposite way: cells cultured in a medium without differentiating factors (i.e., MEM) have the potential for osteogenic differentiation, even without differentiating factors, but only owing to the signals provided by the 3D structure of the MS. Additionally, we investigated the impact of medium composition, i.e., the presence of osteogenic differentiation factors (osteogenic medium, OSG) and addition of medium from osteoclast cell cultures (OSCs). In our approach, we aimed to verify whether factors released by osteoclasts to culture medium participate in intercellular communication between osteoblast cells and impact the osteogenic differentiation process, as suggested by others [28,29].

Our zero hypothesis was that by appropriate design and surface modification of PLGA MS, as well as by assuring suitable conditions of cell culture, we will be to (i) enhance bone cell adhesion, proliferation, and osteogenic differentiation and (ii) obtain material/cell/ECM constructs, by exploring cell ability to remodel and reorganize their surroundings. Such engineered biological constructs produced according to the “bottom-up” approach may be in the future used to create larger tissue units according to the MTE approach and applied in the treatment of bone tissue lesions.

## 2. Results and Discussion

### 2.1. Microstructure and Surface Composition of MS

**Figure 1:**
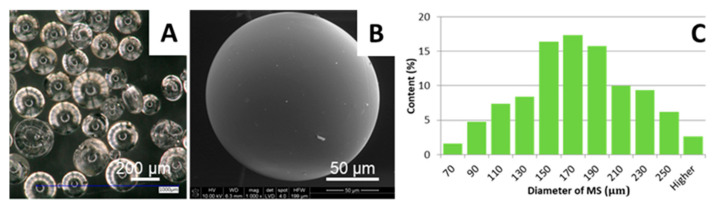
Microstructure of MS observed under optical microscopy (**A**), scanning electron microscopy (**B**), and histogram of the size distribution of MS, *n* = 500 (**C**).

MSs obtained by the emulsification process were spherical and transparent (Figure 1A). Note that each MS shown in Figure 1A resembles a sphere with one “air bubble” in its center. Interestingly, several rays propagate toward the edges of the microsphere. This characteristic appearance is due to the construction of the illuminator of this type of optical microscope, which is made of an O-ring filament. As a result, light is reflected by each microsphere in such a characteristic way, being in fact an artifact. The surface of MS was smooth without pores or other topographical features, as shown by SEM (Figure 1B). MS were regular in shape (shape factor, SF = 1.05 ± 0.06), and their average diameter was 165 ± 47 µm. The histogram presenting the diameter distribution of MS is shown in Figure 1C. The highest percentage of the particles was in the range of 140–200 µm; such size according to the literature is favorable for cell culture [12,13,14,15,16,17].

XPS analysis results (Table 1) show differences in surface chemical composition between MS samples: NaOH-treated MS had lower content of carbon and higher content of surface oxygen than pristine PLGA MS. Detailed O_1s_ spectra (data not presented) showed increased concentration of O–C and O=C groups, suggesting the creation of oxygenated functions after NaOH treatment. Due to the presence of such functional groups on the surface of MS and the presence of carboxyl, amine, and other type groups in PLL and COL molecules, stronger interactions between coatings and MS were facilitated, as found earlier [20].

PLL and COL are built of amino acids and thus contain nitrogen in their structure. The presence of nitrogen peak in the XPS spectra of MS samples after coating with PLL and COL is proof that both components are present on the surface of MS, and the modification procedure was successful.

### 2.2. In Vitro Cell Culture on MS

**Figure 2:**
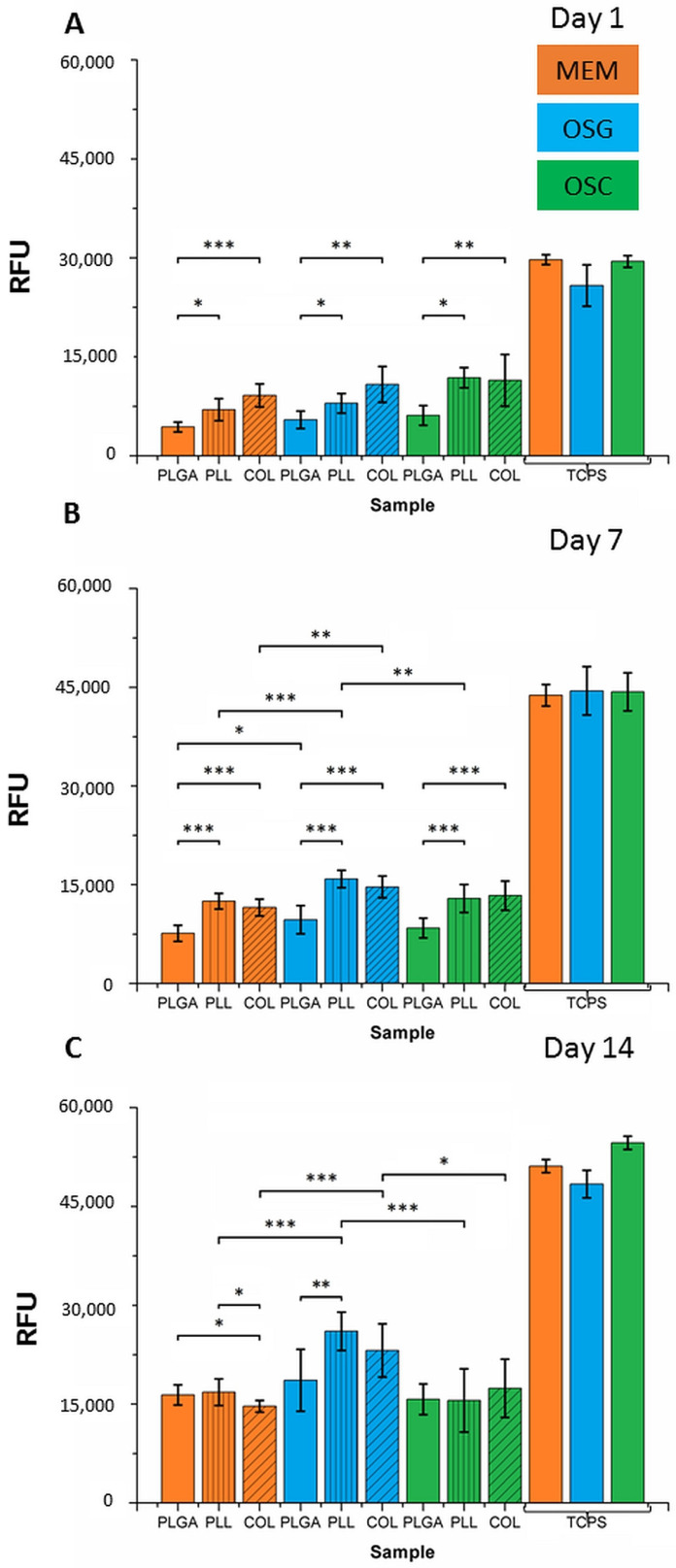
Viability (resazurin assay) of MC3T3-E1 cells cultured for 1 (**A**), 7 (**B**), and 14 (**C**) days on uncoated MS (PLGA) and coated with poly-L-lysine (PLL) and collagen (COL) in different media: MEM, OSG, and OSC, and expressed in relative fluorescence units (RFU). Statistical significance according to one-way ANOVA (* *p* < 0.05, ** *p* < 0.005, *** *p* < 0.001).

Resazurin test was performed on days 1, 7, and 14 to examine the viability of the cells cultured on MS differing in coating type (uncoated, PLL- and COL-coated PLGA MS) and in different media (MEM, OSG, OSC). Cell cultures with MS were performed in anti-adhesive plates in order to prevent cell adhesion on the bottom of the wells but force the cells to seed and adhere to MS. MC3T3 cells were also cultured on oxygen plasma-treated polystyrene plates (TCPS), as a reference (Figure 2).

The cells cultured on TCPS showed increased viability as a function of culture time irrespectively of the type of culture medium: relative fluorescence units (RFU) increased from 30,000 on day 1 to 45,000 on day 7 and 52,000 on day 14. These results demonstrated that MSs do not induce cell toxicity. No significant differences were found between media types for respective cell culture time.

Obtained results showed a very similar trend in every type of medium on day 1: cell viability was the lowest for uncoated MS, then higher for PLL, and the highest for COL. It suggests that the presence of PLL and COL coating on the surface of MS favored cell adhesion and survival. Nevertheless, the most positive effect was observed for COL. There was also a significant difference between PLGA and PLL but not between PLL and COL. There was no difference between different media—cell viability was the same in MEM, OSG, and OSC when compared with the group of the same type of coating. On TCPS cell viability was the same for all media.

Results on day 7 show that significantly higher cell viability was observed on PLL and COL than on uncoated PLGA MS. Cell growth on all MS types in OSG medium was the highest.

On day 14 of cell culture in MEM, the growth of cells on PLGA and PLL was higher than on COL. In OSG medium, enhanced growth was on PLL and COL. PLL- and COL-coated MSs in the OSG medium were the most supportive for cell growth. Uncoated samples exhibited the same growth of cells on their surfaces independently of the type of used medium. OSG medium was found to be the most efficient for MC3T3-E1 cells growth, especially on PLL- and COL-coated MSs.

**Figure 3:**
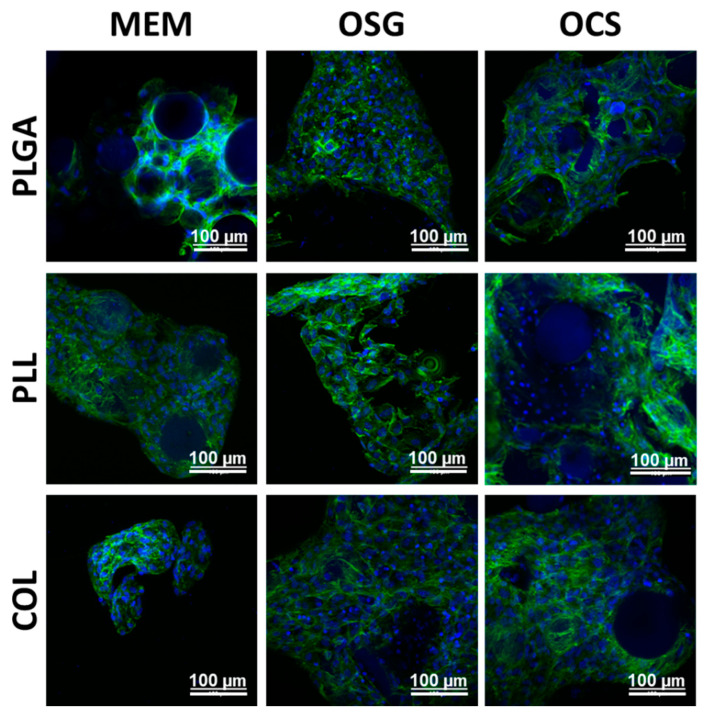
Confocal microscopy results of MC3T3-E1 cells cultured for 7 days on uncoated MS (PLGA) and coated with poly-L-lysine (PLL) and collagen (COL) in different media: MEM, OSG, and OSC; cells after immunofluorescent staining of cytoskeleton fibers (Alexa Fluor 488-Phalloidin) and nucleic acids (DAPI). Scale bar = 100 µm.

Confocal microscopy results (Figure 3) on day 7 show that the cells were well spread, contacting each other and growing on all MSs. The cells were growing on all MS types and formed three-dimensional material–cell constructs. It might be observed that the lowest cell adhesion with the least value was found on uncoated PLGA-MS in MEM condition.

**Figure 4:**
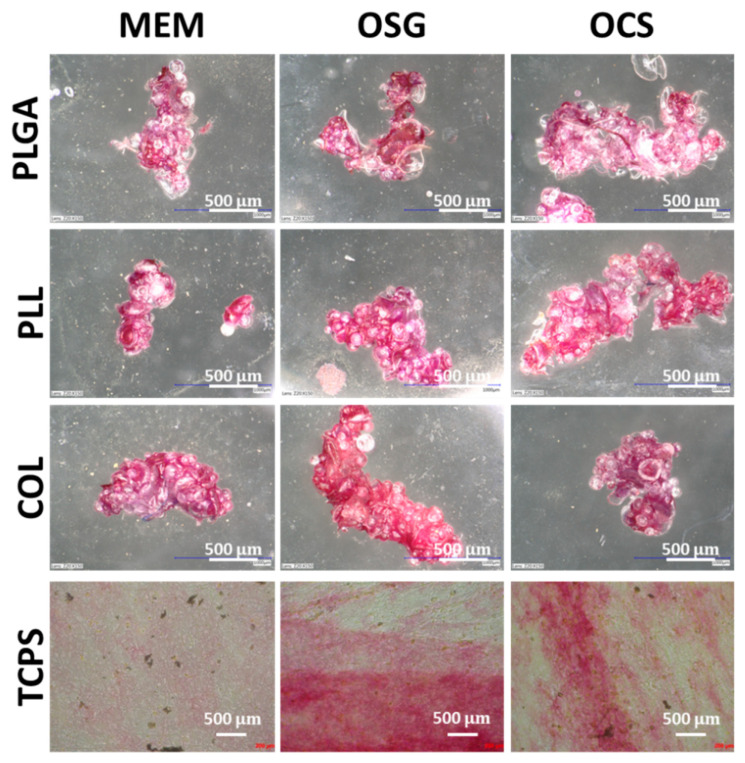
Optical microscopy results of MC3T3-E1 cells cultured for 7 days on uncoated MS (PLGA) and coated wilt poly-L-lysine (PLL) and collagen (COL) in different media: MEM, OSG, and OSC; cells after alkaline phosphatase staining. As a comparison, cells cultured on TCPS are also shown. Cells cultured on all MSs were observed under VHX-900F (Keyence), and on TCPS under Axiovert (Zeiss). Scale bar = 500 µm.

ALP is an early osteogenic differentiation marker [28], which was used to investigate whether MSs have an impact on the cell differentiation process. ALP activity was observed in every type of cell cultured on MSs for 7 days (Figure 4). Cells cultured in reference flat conditions (TCPS) showed enhanced ALP activity only in the media containing osteogenic differentiation factors (OSG), and in conditioned medium obtained from osteoclast cultures (OSC). The only cells cultured in MEM on reference TCPS did not exhibit ALP activity. Additionally, this test showed that the cells on MS were growing in three dimensions. In every well, we observed that there was no single MS despite the fact that on day 1 of the experiment, MSs were dispersed evenly on the whole surface of the well. On day 7 of culture, cells were forming several individual constructs in every well consisting of 10–50 individual MS bound together with the cells and ECM produced by them. The size of obtained constructs in every well was similar independently of the type of MS and medium. The results suggest that these specific, three-dimensional conditions of cells growth also promoted MC3T3-E1 cells early osteogenic differentiation even without the presence of differentiating factors.

**Figure 5:**
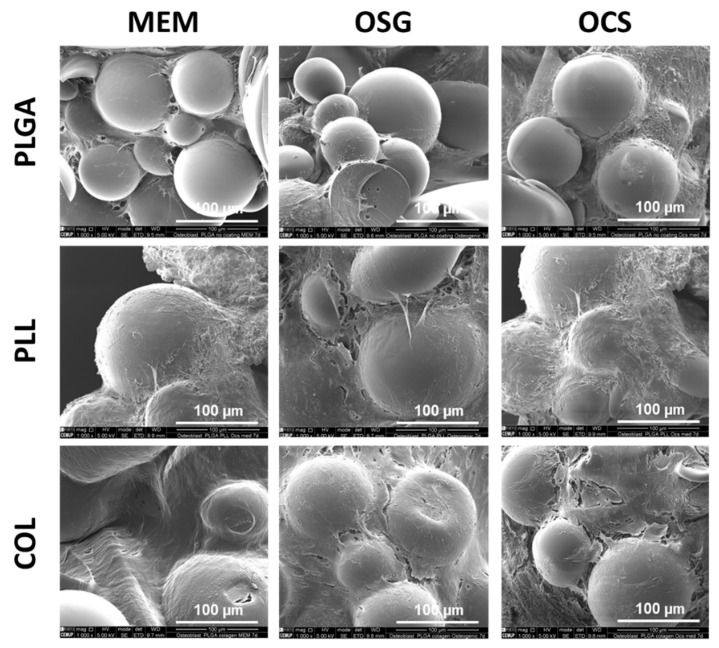
Scanning electron microscopy results of MC3T3-1E cells cultured for 7 days on uncoated MS (PLGA) and coated with poly-L-lysine (PLL) and collagen (COL) in different media: MEM, OSG, and OSC. Scale bar = 100 µm.

SEM results on day 7 (Figure 5) showed that cells did not grow on the whole surface of PLGA MS without any coating, and MSs were embedded only partially in ECM. PLL-and COL-coated-PLGA MSs were better covered with the cells and ECM. This was observed for all culture media tested.

**Figure 6:**
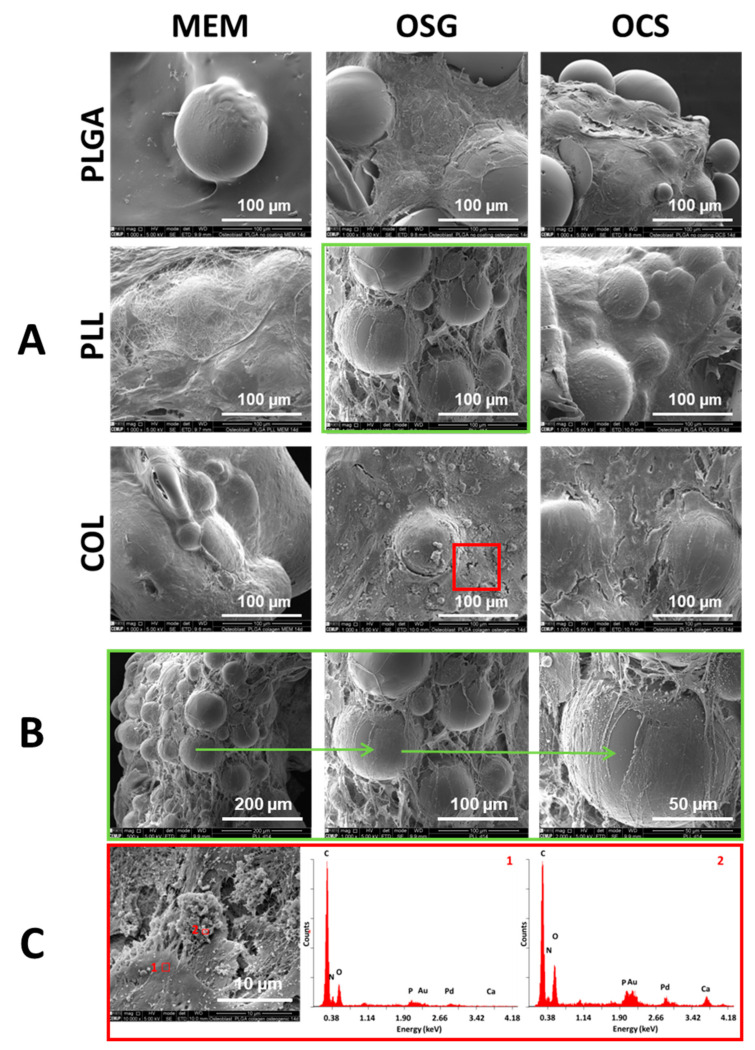
SEM results of MC3T3-1E cells cultured for 14 days on uncoated MS (PLGA) and coated with poly-L-lysine (PLL) and collagen (COL) in different media: MEM, OSG, and OSC (**A**). Detailed microstructure of PLL-modified MS/MC3T3-E1 cells/ECM constructs on day 14 in OSG medium; SEM observation under increasing magnification 500×, 1000×, and 2000× (**B**). SEM image and detailed elemental EDS analysis of the cell (place 1) and of mineral deposits (place 2) present on COL-modified MS/MC3T3-E1 cells/ECM constructs in OSG medium on day 14 (**C**).

Cells cultured on uncoated PLGA MSs after 14 days (Figure 6) still exhibited some differences; MSs were not fully covered with the cells and ECM. PLL and COL were better covered with the cells and ECM, containing numerous fibrous structures visible especially for PLL-coated MSs in MEM and OSG media (Figure 6A).

Obtained constructs in all cases have a form of macroscopic objects made of self-organized MSs, composed of cells and ECM. In Figure 6B, one single construct containing numerous PLL-coated MSs after cell culture for 14 days in OSG medium observed under increasing magnification is shown. Despite the fact that MSs were distributed evenly on the surface of the well on day 1, after 14 days, they were reorganized by the cells and included into a three-dimensional construct bonded by the cells and their ECM.

SEM observation at higher magnification of COL-coated MS in OSG medium on day 14 (Figure 6C) shows areas covered with cells (Figure 6C, point 1) and mineral phase containing calcium phosphate deposits (size around 5–10 µm) (Figure 6C, point 2). EDS analysis of those deposits confirmed the presence of calcium, phosphorus, and oxygen, i.e., elements building calcium phosphates (probably hydroxyapatite). MSs were fully covered with cells and ECM.

**Figure 7:**
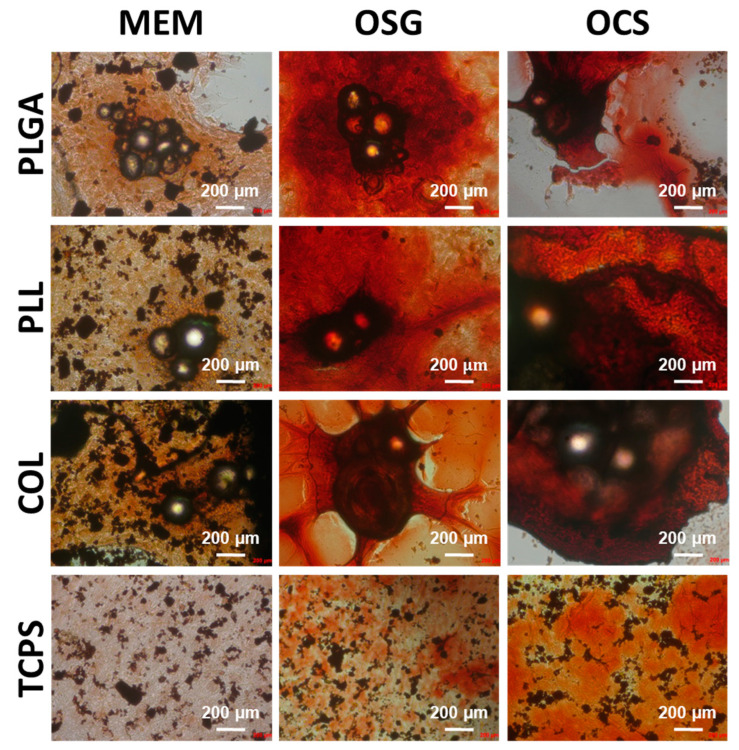
Optical microscopy results of MC3T3-E1 cells cultured for 14 days on uncoated MS (PLGA) and coated with poly(L-lysine) (PLL) and collagen (COL) in different media: MEM, OSG, and OSC; cells after Alizarin red (ARS) staining. As a comparison, cells cultured on TCPS are also shown. Cells observed under Axiovert (Zeiss).

Alizarin red staining of the cells after 14 days of culture was performed to detect calcium deposits and bone module formation, which are considered late osteoblast differentiation factors (Figure 7). For cells cultured in control conditions, i.e., on TCPS, the highest red positive bone module formation was found for osteoblasts cultured in medium harvested from osteoclasts culture (OSC) but lower for medium with osteogenic supplements (OSG); this was not the case for cells cultured in MEM. A similar effect showing enhanced bone module formation was observed by Zhang et al. [30] on MC3T3 cells cultured on glass control and titanium surfaces in a medium collected from osteoclast culture. Our results suggest that MC3T3-E1 exhibited osteogenic potential and that cytokines released by osteoclasts have an anabolic effect on them.

When cells were cultured on MSs, the highest bone module formation was observed when the cells were maintained in media with osteogenic differentiation factors (OSG, OSC). Alizarin red intensity was much higher, as compared to the cells cultured in OSG and OSC on the control flat substrate—TCPS.

The presence or absence of coating on MSs had no impact on the mineralization process. The above results show that MSs created an environment that promoted MC3T3-E1 early osteogenic differentiation, but late osteogenic differentiation (mineralization) was affected to a lower extent. Our findings may suggest that supplementation with osteogenic differentiation molecules should be considered at longer time points of cell culture on MSs.

Our results clearly show that adsorption of cell-adhesive molecules (PLL, COL) on PLGA MSs enhances bone cell adhesion and proliferation. Early osteogenic differentiation is supported by culturing cells on MSs in antiadhesive plates and does not require supplementation with osteogenic differentiation factors. Interestingly, this is not the case for longer cell culture time, when late osteogenic differentiation is expected. As a result, by providing the cells with 3D-supporting structure at micrometric level (PLGA MS) with appropriate surface properties (PLL, COL coating), and by assuring optimal cell culture conditions (antiadhesive plate, cell culture media with defined composition), it is feasible to produce MS/cell/ECM constructs. Such engineered biological constructs are created by the cells themselves, in line with the knowledge that the cells are capable of reorganizing an adjacent microenvironment to form tissue.

Our study is a proof of concept that by providing the cells with proper microenvironment and conditions, it is feasible to mimic tissue modules creation in vitro. Such modules might be further assembled into larger blocks and potentially used in the treatment of bone tissue defects according to the “bottom-up” MTE approach.

## 3. Materials and Methods

### 3.1. MS Manufacturing and Modification

All chemicals were from Sigma-Aldrich unless stated otherwise. MSs were obtained via oil-in-water emulsification. PLGA (85:15, M_n_ = 100 kDa, M_w_ = 210 kDa, produced at the Center of Polymer and Carbon Materials, PAS, Zabrze, Poland) was dissolved in dichloromethane (DCM, Avantor Performance Materials) at a concentration of 7.5% and acted as oil phase. The water phase was prepared by dissolving poly(vinyl alcohol) (PVA, Mowiol^®^ 4–88, M_W_ ca. 31 kDa) in ultra-high-quality water (produced by UHQ-water, Purelab, UK) at a concentration of 1%. Then, 1 mL of oil phase was added to 50 mL of water phase stirred by magnetic stirrer (1000 rpm, JeioTech, Model MS-52M) and left for 24 h for DCM evaporation. Afterward, MSs were washed multiple times with UHQ water and dried at 37 °C for 24 h. The detailed method of manufacturing and the results showing the impact of selected processing parameters on the size and polydispersity of MSs are described elsewhere [31].

MSs were treated with 0.1 M NaOH (100 mg MS per 10 mL NaOH, 30 min, shaken), washed three times with UHQ water, and dried. Afterward, 40 mg of such MSs was incubated in 2 mL 100 μg/mL collagen solution (type I, human recombinant, expressed in Nicotiana tabacum, Collagen^TM^) for 24 h. Another portion of MSs (40 mg) was incubated in 100 μg/mL poly-L-lysine (PLL, MW 150–300 kDa) for 24 h. Then, samples were washed with UHQ–water three times and dried at 37 °C for 24 h.

### 3.2. Optical and Scanning Electron Microscopy Characterization of MS

Optical microscopy observations of MSs were performed with Axiovert, Zeiss, and Keyence VHX-900F microscopes. MS diameter was measured in two perpendicular directions of each MS to determine average diameter and shape factor (SF) calculated by dividing higher value of diameter by lover value of the diameter of each MS (*n* = 500). MSs were also observed under a scanning electron microscope (SEM, Nova NanoSEM 200, Hillsboro, OR, USA). Prior to observation, they were coated with a 20 nm carbon layer to make them conductive.

### 3.3. X-ray Photoelectron Spectroscopy Analysis of MS

The chemical elemental surface composition was determined by an SSI 100/206 photoelectron spectrometer from Surface Science Instruments (USA) equipped with a monochromatized microfocused Al X-ray source (powered at 20 mA and 10 kV). The pressure in the chamber was around 10^−6^ Pa. To perform XPS analysis, MSs were fixed with double-sided tape onto small brass troughs of 6 mm diameter that were placed on a ceramic carousel. The angle between the surface normal and the axis of the analyzer lens was 55°. The analyzed area was approximately 1.4 mm^2^, and the pass energy was set at 150 eV. The full width measured at half maximum (FWHM) of the Au_4f7/2_ peak for a clean gold standard sample was about 1.6 eV. A flood gun set at 8 eV and a Ni grid placed 3 mm above the sample surface were used for charge stabilization. The following sequence of spectra was recorded: survey spectrum, C_1s_, O_1s_, N_1s_, and C_1s_ again to check the stability of charge compensation with time. The binding energy scale was referenced by setting the C–(C,H) component of the C_1s_ peak at 284.8 eV.

### 3.4. In Vitro Cell Culture on MS

Biological tests were performed with MC3T3-E1 mouse calvaria preosteoblast cells (obtained from the European Collection of Cell Cultures-ECACC) at passage 11. Three types of media for cell cultures were used: MEM, i.e., basal complete medium (α-modified minimum essential medium, supplemented with 10% of FBS and 1% of penicillin/streptomycin, Gibco); OSG, i.e., osteogenic medium (complete MEM with the addition of the following differentiating factors: 50 µM ascorbic acid and 10 µM sodium β-glycerophosphate); OSC, i.e., osteoclast conditioned medium retrieved from mouse osteoclast cultures at day 4 of osteoclast differentiation (as described in [32]) at ratio 1:1. Prior to cell seeding, 100 µL of MS suspension (uncoated, coated with PLL, or coated with COL) in respective cell culture medium (at a concentration of 10 mg of MS per 1 mL medium) was poured into wells of 96-well anti-adhesive polystyrene plate (Greiner 96-well plates). Additionally, a reference plate made of TCPS (tissue-culture treated plates, FALCON^®^ 96 Well) without MS was prepared. Afterward, the plates were left under a laminar flow chamber for 15 min under UV irradiation as a sterilization process. Afterward, 15 × 10^3^ cells suspended in 100 µL of respective medium were added to every well and cultured at 37 °C under 5% CO_2_. At the time of medium change (3, 7, and 10 days of culture), only half of the volume was removed (to avoid detaching MS/cells constructs deposited in the bottom of the wells) and refreshed with the respective medium.

### 3.5. Cell Viability

Resazurin assay tests were performed after 1, 7, and 14 days of culture by the addition of 20 µL resazurin solution to every well plate to achieve a final concentration of 10%. Subsequently, samples were placed in an incubator (5% CO_2_, 37 °C) for 3 h, and after incubation 100 µL solution was aspired, added to black test plates (96-well black microplate, polystyrene, Greiner Bio-One), and fluorescence was measured (at excitation 530–560 nm and emission 590 nm) using a microplate reader spectrophotometer (Bioteck Plate Reader, Synergy MX) and expressed in relative fluorescence units (RFU).

### 3.6. Alkaline Phosphatase Assay

For ALP staining, the samples after 7-day cell culture were washed with distilled water, fixed with 4% paraformaldehyde (PFA) for 10 min at room temperature, and then washed again with deionized water. Then, 100 µL of Naphtol AS-MX phosphate/Fast Violet B salt solution was added to each well and incubated at room temperature in the dark for 45 min, and the samples were washed twice with deionized water. After drying, samples were observed under optical microscopes (Axiovert, Zeiss, Oberkochen, Germany and Keyence VHX-900F, Mechelen, Belgium).

### 3.7. Mineralization Assay

For Alizarin red staining, the culture medium was removed, and wells were washed with phosphate-buffered saline (PBS). Cells were fixed with 200 µL ice-cold 70% ethanol for 1h at −20 °C and then air-dried. Subsequently, 150 µL of 2% alizarin red (pH = 4.2) was added and maintained for 15 min at room temperature. After that, the samples were washed with deionized water and analyzed under optical microscopes and with the same conditions, as described above.

### 3.8. Cell Morphology Assay—Confocal, Optical, and Scanning Electron Microscopy

Samples for laser confocal scanning microscopy (LCM, widefield inverted microscope DMI6000 FFW; Leica Microsystems) were washed with PBS, and then cells were fixed with 100 µL 4% PFA for 10 min at room temperature and washed with PBS. Subsequently, the cells were permeabilized in ice-cold 0.1% Triton X solution for 5 min, blocked with 1% BSA for 1 h at 37 °C, incubated in Alexa Fluor 488-Phalloidin (1:150) for 1 h in dark, and then incubated for 5 min in DAPI at room temperature. Between every step, washing with PBS was conducted. Images were captured with an LCM equipped with LAS X software (Leica Microsystems) at the i3S advanced light microscopy unit. For SEM studies, the samples from days 7 and 14 of cell culture were washed with PBS, and then the MSs were moved to a new plate and kept in 2.5% cacodylate buffer in sodium cacodylate at room temperature for 30 min and then washed with cacodylate buffer. Subsequently, samples were washed with aq. ethanol solutions with increasing concentration, starting from 50%, increasing by 10% to absolute ethanol, and kept in every concentration for 10 min. Then, 100 µL of hexamethyl disilane was added and left to evaporate overnight. The samples were coated with Au/Pd thin film by sputtering using the SPI Module Sputter Coater. The SEM/EDS analysis was performed using the high-resolution Schottky scanning electron microscope with X-Ray microanalysis and rectangular electron diffraction pattern analysis (Quanta 400FEG ESEM/EDAX Genesis X4M, Hillsboro, OR, USA).

### 3.9. Statistical Analysis

The statistical analyses of the obtained data were performed using a one-way analysis of variance (one-way-AVOVA) followed by Tukey’s post hoc test. The assumptions of normal distribution and equal variance were verified using the Shapiro–Wilk and Levene median tests, respectively (*p*-value < 0.05). The analyses were performed using SigmaPlot 12.3 software (Systat Software, Inc., San Jose, CA, USA). The results are presented as mean ± standard deviation (SD) unless stated otherwise. Statistical significance was considered for *p* < 0.05 (*), *p* < 0.01 (**) and *p* < 0.001 (***).

## 4. Conclusions

PLGA MSs coated with PLL and COL enhanced adhesion and proliferation of preosteoblast MC3T3-E1 cells, as shown by cell metabolic activity test and microscopic observation after fluorescence staining of the cytoskeleton and nuclei.

The results indicate that 3D MS/cell/ECM constructs were created spontaneously in static conditions owing to cell auto-assembly facilitated by the use of anti-adhesive cell culture plates. ECM creation was enhanced on PLL- and COL-coated MSs, as compared to those MSs without coating.

Early osteogenic differentiation of the cells, as shown by ALP activity on day 7, was observed on all types of MSs in all used media types tested. Interestingly, cells did not exhibit ALP activity, when they were cultured in MEM on control TCPS. It means that cell culture on the MS enhances their early osteogenic differentiation and creation of MS/cell/ECM constructs.

Late osteogenic differentiation, as shown by the presence of mineral deposits visible under SEM and analyzed by EDS, as well as Alizarin red staining on day 14, was dependent on medium composition, i.e., the presence of soluble differentiation factors.

Moreover, MC3T3-E1 cell culture in medium collected from osteoclasts cultures was found to have similar osteogenic activity as typical osteogenic differentiation medium. Further studies are needed to assess whether factors released by osteoclasts to culture medium may participate in intercellular communication between osteoblasts and impact their differentiation process.

To summarize, we found that preosteoblast MC3T3-E1 cells cultured in a medium without differentiating factors (i.e., MEM) have the potential for osteogenic differentiation, owing to the signals provided by the 3D structure of the MSs and their assemblies created by cultured cells and bounded with ECM. However, the impact of the 3D structure of microspheres and their assemblies on late osteogenic differentiation should be supported by soluble osteogenic differentiation molecules added to the cell culture medium. Our findings pave a way for the application of surface-modified PLGA MS as substrates to obtain self-assembled MS/cell/ECM modules supporting bone tissue regeneration particularly in the treatment of critical size tissue defects.

## Figures and Tables

**Table 1:** Surface chemical composition (except hydrogen) of MS measured by XPS.

MS Sample	C (at. %)	O (at. %)	*n* (at. %)
PLGA pristine	65.27	34.72	^bdl^
PLGA after NaOH treatment	58.18	41.81	^bdl^
PLL	59.7–64.4	35.2–39.8	0.3–0.5
COL	58.0–60.7	29.2–39.2	1.0–3.9

^bdl^ below the detection limit, element percentage ranges for samples coated with PLL and COL, and analyses of samples from two batches.

## Data Availability

Not applicable.
